# *Tg*ROP18 targets IL20RB for host-defense-related-STAT3 activation during *Toxoplasma gondii* infection

**DOI:** 10.1186/s13071-020-04251-7

**Published:** 2020-08-07

**Authors:** Ling Kong, Dan Jiang, Cheng He, Jing Xia, Haixia Wei, Lijuan Zhou, Hongjuan Peng

**Affiliations:** Department of Pathogen Biology, Guangdong Provincial Key Laboratory of Tropical Disease Research, School of Public Health, Southern Medical University, Guangzhou, 510515 Guangdong Province China

**Keywords:** *Toxoplasma gondii*, ROP18, IL20RB, STAT3, Proinflammatory immunity

## Abstract

**Background:**

*Toxoplasma gondii* is an opportunistic protozoan infecting almost one-third of the world’s population. *Toxoplasma gondii* rhoptry protein 18 (*Tg*ROP18) is a key virulence factor determining the parasite’s acute virulence and is secreted into host cells during infection. We previously identified the interaction of *Tg*ROP18 and host cell immune-related receptor protein IL20RB, and observed the activation of STAT3 in human keratinocytes (HaCaT) cells infected by the *rop16* knockout RH strain, though *Tg*ROP16 is regarded as being responsible for host STAT3 activation during *T. gondii* invasion. Therefore, we hypothesize *Tg*ROP18 can activate host STAT3 through binding to IL20RB.

**Methods:**

CRISPR-CAS9 technology was used to generate the ROP16 and ROP18 double knockout RH strain, RH-∆*rop16*∆*rop18*. SDS-PAGE and western blot were used to detect STAT3 activation in different HaCaT cells with high endogenous IL20RB expression treated with *T. gondii* tachyzoites infection, recombinant ROP18, or IL-20. FRET and co-immunoprecipitation (Co-IP) was used to detect the protein-protein interaction.

**Results:**

We observed that *Tg*ROP18 was involved in a synergic activation of the host JAK/STAT3 pathway together with *Tg*ROP16 in human HaCaT cells infected with *T. gondii* or treated with recombinant *Tg*ROP18 protein, stimulating host proinflammatory immune responses such as expression of TNF-α. The effect of recombinant ROP18 on STAT3 phosphorylation was presented in a dose-dependent manner. Additionally, *Tg*ROP18 was identified to target IL20RB on its extracellular domain. When we treated different cell lines with the recombinant ROP18, STAT3 phosphorylation could only be observed in the cells with endogenous IL20RB expression, such as HaCaT cells.

**Conclusions:**

These findings indicate that *Tg*ROP18-IL20RB interaction upon *T. gondii* invasion was involved in STAT3 activation, which is associated with host cell defense.
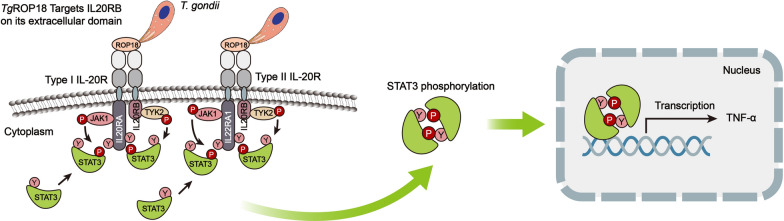

## Background

*Toxoplasma gondii* is an obligate intracellular parasite of the phylum Apicomplexa, which resides in the nucleated cells of warm-blooded vertebrates [1, 2] It is reported that approximately one-third of the world’s population are chronically infected with *T. gondii* [3, 4]. *Toxoplasma gondii* infection in immunocompetent individuals is usually asymptomatic, whereas, in immunosuppressed individuals or in primary infection during pregnancy, it may cause severe toxoplasmosis and even death [5–7].

The apical complex of the apicomplexan organism, consists of conoid and apical organelles, and is responsible for host cell penetration and the establishment of intracellular parasitism. The apical organelles, including rhoptries, micronemes and dense granules, are characteristic secretory vesicles which discharge proteins involved in the cell invasion [8]. Host cell invasion by *T. gondii* is a rapid but complicated process [9], including cell membrane attachment, protein secretion, moving junction formation and inclusion [10–12]. Host cell activities, including innate and adaptive immune responses, nutrient metabolism, cytoskeletal rearrangement, and apoptosis, are manipulated during *Toxoplasma* tachyzoite invasion [13–16]. During invasion, many of the effectors are injected into host cells by *T. gondii*, including rhoptry proteins (ROPs), dense granule proteins (GRAs), and microneme proteins (MICs) [17, 18]. These injected ROPs, such as ROP16 and ROP18, have been known to activate host transcription factors, such as signal transducer and activator of transcription (STAT), to inhibit host immune responses [19–21]. Moreover, a previous study has shown that a large number of rhoptry proteins are injected into cells that are not infected, and result in transcription factor activation comparable to that of infected cells [18]. Meanwhile, *T. gondii* modulates host immune responses to maintain a balance between pro-inflammatory and anti-inflammatory reactions to allow its successful parasitism, and thereby to establish a long-term (chronic) infection [22].

*Tg*ROP18 is a serine/threonine kinase secreted from the rhoptry during parasite invasion, and is known as a critical contributor to the virulence of *T. gondii* [23]. Detectable *Tg*ROP18 is reported to be only secreted by type I and type II strains, but not by type III strains. *Tg*ROP18 can suppress the differentiation of host neural stem cells [24], inhibit the host NF-κB pathway by promoting p65 degradation in HEK 293T cells [25], and target phosphorylation of IFN-inducible small GTPases (IRGs) in murine cells or ATF6β degradation in human cells for disarming the host’s innate and adaptive immunity [26, 27].

Type I interleukin-20 receptor (IL-20R) is a heterodimeric receptor that mediates IL-19, IL-20 and IL-24 signaling, and is composed of two subunits, IL20RA and IL20RB; type II IL-20R mediates IL-20 and IL-24 signaling and is composed of IL22RA1 and IL20RB [28, 29]. Both types of receptors are expressed in various non-immune tissues, especially in epithelial cells [30]. Upon activation, IL20RB phosphorylates JAK1 and activates the JAK/STAT3 pathway; phosphorylated STAT3 then forms homodimers and translocate to the nucleus to initiate transcription of downstream proinflammatory cytokines, including tumor necrotic factor -α (TNF-α) and monocyte chemotactic protein 1 (MCP-1), by binding to STAT-responsive promoter elements [31, 32]. It is well documented that STAT3 is the dominant transcription factor activated by both type I and type II IL20 receptors [31].

It is generally recognized that *Tg*ROP16 is dominantly responsible for host STAT3 activation during *T. gondii* invasion [33, 34], and no study has been conducted to show if ROP18 functions on STAT3 activation. In our study, we were surprised to observe the activation of STAT3 in human keratinocyte (HaCaT) cells infected by the *rop16* knockout RH strain (RH-∆*rop16*). Combined with the finding of *Tg*ROP18-human IL20RB interaction in our previous research [35], we hypothesized that *Tg*ROP18 may activate STAT3 through binding with IL-20R to activate the JAK/STAT3 pathway. The HaCaT cell is a type of epithelial cell expressing both type I and type II IL-20 receptors in high levels, therefore, it was used as a cell model in this research.

## Methods

### Cell culture and *T. gondii* propagation

Human foreskin fibroblasts (HFF), African green monkey kidney cells transformed by SV40 (COS-7), human embryonic kidney 293T (HEK 293T) cells, and human keratinocytes (HaCaT) cells were bought from the American Type Culture Collection (ATCC, Manassas, USA), and cultured in Dulbecco’s modified Eagleʼs medium (DMEM; Gibco/Invitrogen, Carlsbad, USA) containing 10% (v/v) FBS (Gibco/Invitrogen) and 1% gentamicin (10 mg/ml; Invitrogen, Carlsbad, USA) at 5% CO_2_ and 37 °C. For fluorescence resonance energy transfer (FRET) experiments, COS-7 cells were grown on coverslips in 12-well plates (Corning, Corning, USA). For pSTAT3 detection, HaCaT or COS-7 cells were seeded on 6-well plates and grown to 100% confluence, and then cultured in serum-free medium overnight before treatment with IL-20 (R&D Systems, Minneapolis, USA), recombinant GST-ROP18, or *T. gondii* infection.

The tachyzoites of CEP, CEP-*Tg*ROP18I, and CEP-*Tg*ROP18II were a generous gift from Jon Boyle at the University of Pittsburgh, USA. The tachyzoites of *T. gondii* RH strain, *rop16* knockout strain *(*RH-∆*rop16*), *rop18* knockout strain (RH-∆*rop18*), and *rop16+rop18* double knockout strain (RH-∆*rop16*∆*rop18*) were propagated in HFFs with DMEM supplemented with 1% FBS, as well as the CEP and its mutant strains. Before experiments, the tachyzoites cultured in HFFs were purified by passing through a 3 µm filter after the host cells were ruptured or syringed broken, and then counted.

### Mammalian eukaryotic expression

The cDNA of IL20RB on pDONR was generously gifted by Professor Wen-Bin Ma at the School of Life Science, Sun Yat-Sen University, China. The cDNA of IL20RB was amplified by PCR using *Pfu* DNA polymerase (TransGen Biotech, Beijing, China), to add the HA tag on its C-terminus and incorporate *Sal*I and *Sac*II restriction sites on the 5′- and 3′-ends, respectively. To perform FRET and co-immunoprecipitation (Co-IP) assays, IL20RB-HA, and its extracellular and intracellular domain was then cloned into pEYFP-C1 to form pEYFPC1-IL20RB-HA, pEYFPC1-IL20RB-Extr-HA (Extr, extracellular domain of IL20RB), and pEYFPC1-IL20RB-Cyt-HA (Cyt, intracellular domain of IL20RB). The construction of pCFPN1-ROP18-3×FLAG has been described in detail elsewhere [16]. All the primers used are shown in Additional file 1: Table S1.

### CRISPR-CAS9 mediated generation of RH*-∆rop16∆rop18* double knockout strain

RH-Δ*rop16* was constructed in our previous research. To disrupt the *rop18* gene in the RH-Δ*rop16* strain, a recombinant CRISPR plasmid, pSAG1::CAS9-U6::sgROP18, which contains an sgRNA specifically targeting the site very close to and downstream from the start codon of *rop18* gene, was constructed, using a Q5^®^ Site-Directed mutagenesis kit (NEB, Beverly, USA) following the published protocol [36]. A chloramphenicol-resistance gene (chloramphenicol acetyltransferase, CAT) was used as the screening marker. The homologous recombination plasmid (pBlue-5′-3′-ROP18-homo-CAT) was constructed as follows (the procedure is also shown in Additional file 2: Figure S1a); a 991-bp fragment and a 965-bp fragment homologous to the upstream and downstream of the gRNA targeted site of the *rop18* gene, were respectively subcloned to the 5′- and 3-end of the CAT gene cassette on pBlue-script SK II (−) plasmid [16]. Subsequently, these two recombinant plasmids were co-transfected into RH-Δ*rop16* tachyzoites by electroporation. The transfected tachyzoites were used to infect HFF cells, and cultured in chloramphenicol free DMEM complete medium for 48 h. To screen the recombinants, the tachyzoites were cultured in the DMEM complete medium containing 20 mmol/l of chloramphenicol for 4 passages. Finally, the double-knockout RH-Δ*rop16*Δ*rop18* tachyzoites were screened through monoclonal screening, and identified by PCR. All the PCR primers used for homologous template amplification, plasmid construction, and recombinant identification are shown in Additional file 1: Table S1.

### Detection of STAT3 activation in different *T. gondii* tachyzoites infected or IL-20 treated HaCaT cells

#### Cell preparation

(i) *HaCaT cell infection with RH-WT, RH-Δrop16, RH-Δrop18, and RH-Δrop16Δrop18 strains.* HaCaT cells were cultured in 6-well plates to 100% confluence. After the cells were washed 3 times with PBS and switched to serum-free medium and starved overnight, RH-WT, RH-Δ*rop16*, RH-Δ*rop18*, and RH-Δ*rop16*Δ*rop18* strains were used to infect the cells by one well for each, at a multiplicity of infection (MOI) of 10 for 30 min; another well was not infected for the control.

(ii) *HaCaT cell treatment with IL20, or infection with RH-WT and RH-Δrop18 strains.* HaCaT cells were cultured in 6-well plates to 100% confluence. After the cells were starved overnight, two wells of cells were treated with 400 ng of IL-20 for 30 min and 24 h, respectively; 2 wells were infected with RH-WT at a MOI of 10 for 30 min and 24 h, respectively, and 2 wells were infected with RH-Δ*rop18* under the same conditions; another well was not infected for the control.

(iii) *HaCaT cell treatment with recombinant GST-ROP18 and GST at different amounts.* The construction of the prokaryotic expression vector and expression of GST-ROP18 and GST have been described in detail previously [16]. The *Escherichia coli* expressed GST-ROP18 and GST proteins were purified using the GST fusion protein purification kit (GenScript Inc., Beijing, China) following the manufacturer’s instructions. The concentration of the purified protein was detected using a NanoDrop 2000 (Thermo Fisher Scientific, Waltham, USA). HaCaT cells were cultured in 6-well plates to 100% confluence. The cells were starved overnight, and treated with 0.3, 0.6, 1.2 and 2.4 mg of GST and GST-ROP18, separately, one well for each treatment. Another well was treated with 10 µl of elution buffer (20 mM glutathione, 50 mM Tris-HCl, pH 8.0) for the control.

(iv) *HaCaT cell treatment with IL-20, GST-ROP18 and GST, and detection of the co-localization of ROP18 and IL20RB on HaCaT cell membrane COS-7.* HaCaT and HFF cells were grown in 6-well plates to 100% confluence. The serum starved cells were treated with IL-20 (200 ng), recombinant GST (2 μg), or recombinant GST-ROP18 (2 μg) for 30 min, and elution buffer (50 mM Tris-HCl 8.0 with 20 mM glutathione, pH 8.0) as a negative control, with one well for each treatment.

(v) *HaCaT cell infection with type III CEP, CEP-TgROP18I, and CEP-TgROP18II strains.* HaCaT cells were grown in 6-well plates to 100% confluence, cells were starved overnight and infected with CEP, CEP-*Tg*ROP18I, and CEP-*Tg*ROP18II at a MOI of 10 for 30 min, with one well for each infection. Another well was uninfected (control).

#### Detection of STAT3 activation

The culture medium was aspirated, the cells were then washed with PBS 3 times, and the unrecruited parasites were washed off. The cells were harvested and lysed. Fifty milligrams of total protein was subjected to SDS-PAGE and western blot to detect activation of STAT3 (phosphorylation at Y705 or S727). β-actin was detected as the loading control for the whole cell lysate. Densitometric quantitation of each band in A was applied using Image J. The optical density of Stat3-pY705 and Stat3-pS727 was normalized to the density of the total STAT3, and divided by the uninfected group whose normalized value was set as 1.

#### Statistical analysis

All experiments were performed at least in triplicate. Data are presented as means ± SD of 3 independent experiments. Statistical significance was determined using the Kruskal-Wallis H-test with Bonferroni correction or an independent t-test. Values of *P < 0.05, **P < 0.01 and ***P < 0.001 were defined as statistically significant.

### Western blot analysis and co-immunoprecipitation

COS-7 cells transfected with recombinant plasmid or HaCaT cells infected with parasites were lysed in lysis buffer (Beyotime Biotechnology, Shanghai, China) containing a protease inhibitor cocktail (Thermo Fisher Scientific) or/and a phosphatase inhibitor cocktail (TransGen Biotech). The cell lysates were centrifuged at 14,000×*g* for 5 min at 4 °C. The supernatants (50 mg of total proteins) were separated by SDS-PAGE and then transferred to PVDF membranes using Trans-Blot SD semi-dry electrophoretic transfer (Bio-Rad, Foster, USA). Then, the PVDF membranes were blocked in PBS or TBS containing 5% bovine serum albumin (BSA; Sigma-Aldrich, Darmstadt, Germany) and 0.05% Tween-20. After blocking, the PVDF membrane was incubated with primary antibodies overnight at 4 °C. Then, membranes were probed with secondary antibodies conjugated with horseradish peroxidase (HRP) for 2 h. The proteins of interest were visualized by luminescence generated using Clarity^TM^ western ECL substrate (Bio-Rad) and imaged using a ChemiDoc Touch Imaging System (Bio-Rad.).

For co-immunoprecipitation (Co-IP), the supernatants of cell lysates were incubated with 4 μg of anti-HA tag antibody at 4 °C for 1 h. Then, Protein A-Agarose (Santa Cruz Biotechnology) was added to the mix and incubated for 12 h with gentle rotation at 4 °C. The agarose beads were collected by centrifugation for 5 min at 1000×*g* at 4 °C and washed 4 times with PBS. Then the immunoprecipitates were resuspended in SDS-PAGE sample buffer (Takara, Otsu, Japan) and boiled for 10 min followed by soaking immediately in ice water. The boiled samples were centrifuged at high speed, the supernatant was then loaded for SDS-PAGE, then subjected to western blot analysis with the antibodies described below.

### Fluorescence resonance energy transfer (FRET) experiment

COS-7 cells were seeded on coverslips in a 12-well plate, and grown to 80% confluence. The experimental group was co-transfected with pCFPN1-ROP18-3×FLAG and pEYFPC1-IL20RB-HA for 48 h. The mono-fluorescence groups were transfected with pCFPN1-ROP18-3×FLAG or pEYFPC1-IL20RB-HA separately for adjustment of the fluorescence base line of donor (eCFP) and acceptor (eYFP). The negative control group was co-transfected with pECFPN1 and pEYFPC1, and the positive control group was transfected with pEYFP-ECFP. The co-localization of ECFP-ROP18 with EYFP-IL20RB was observed under a FluoView FV1000 confocal microscope (Olympus, Tokyo, Japan), and the FRET efficiency and intermolecular distance were calculated by sensitized emission (SE) program. The detection was repeated in different cells three times. The difference between groups was analyzed with the t-test using SPSS software (**P < 0.01).

### Reverse transcription PCR (RT-PCR) and quantitative reverse transcription PCR (qRT-PCR)

The cells were starved overnight before infection and detection. The transcription level of IL20RB in COS-7, HEK293T, HFF, and HaCaT cells was evaluated by RT-PCR. The transcription levels of IL20RB, IL22RA1, and TNF-α in infected cells were analyzed by qRT-PCR. The normal HaCaT cells, and the cells infected with RH-WT or RH-∆rop18 at a MOI of 10 for 10, 20 and 30 min, were harvested separately. The transcription level of iNOS, IL20, IL22, and IL10 was detected by qRT-PCR. All the PCR primers are shown in Additional file 1: Table S1. The translation level of IL10 was detected by western blot.

Total RNA isolation from infected or uninfected cells was performed using Trizol^®^ reagent (Ambion, Foster, USA), and genomic DNAs were immediately removed by Turbo DNAase (Invitrogen). cDNAs were generated with the RevertAid First Strand cDNA Synthesis Kit (Thermo Fisher Scientific). PCR was conducted using *Pfu* DNA polymerase (TransGen Biotech), and qRT-PCR was performed using SuperReal PreMix plus (Tiangen Biotech, Beijing, China). All of the procedures were performed following the manufacturer’s instructions.

Gene-specific primers are shown in Additional file 1: Table S1. Each experiment was performed in triplicate and the reactions were conducted in a Bio-Rad Applied Biosystems 7500 qPCR system (Bio-Rad). The relative transcription level of each gene was normalized to the mRNA of an internal control gene, GAPDH. The transcription levels of the target genes were compared by the change-in-quantification cycle (∆∆Cq) method.

### Detection of the co-localization of ROP18 and IL20RB on HaCaT cell membrane

HaCaT cells were grown on coverslips in 12-well plates to 100% confluence. The overnight serum starved cells were treated with recombinant GST (1 μg), or recombinant GST- ROP18 (1 μg) for 30 min, and then fixed in 300 μl 4% paraformaldehyde. The cells were then permeabilized with 0.5% Triton X-100/PBS for 10 min, then washed with PBS 3 times, 5 min for each wash. The cells were blocked with 500 μl 10% BSA for 1 h at room temperature, followed by incubation with rabbit anti-IL20RB and mouse anti-GST monoclonal antibody in 10% BSA overnight at 4 °C. After washing with PBS 3 times, 5 min for each wash, the cells were incubated with the secondary antibodies Alexa Fluor 488 goat anti-rabbit IgG and Alexa Fluor 594 goat anti-mouse IgG in 10% BSA for 1 h at room temperature in the dark. The cells were washed with PBS for 3 times, 5 min for each wash. The coverslips were mounted with DAPI mounting oil and observed under a fluorescence microscope (Nikon eclipse Ni; Nikon, Tokyo, Japan) with a 100× objective lens (NA 1.40).

### Antibodies

The primary antibodies used for for western blot: the mouse poly-clonal Ab anti-*Tg*ROP18 (1:1000) was raised in our laboratory; mouse mAb anti-*Tg*SAG1 (1:1000), rabbit anti-HA tag (1:5000), rabbit mAb anti-stat3 (phosphor Y705, 1:200,000), rabbit mAb anti-stat3 (phosphor S727, 1:5000), and rat mAb anti-IL20RB (1:1000) were purchased from Abcam Pharmatech, Inc, (Westlake Village, UK) . Mouse mAb anti-GST (1:2000), mouse mAb anti-DDDDk (1:2000), and rabbit polyclonal Ab anti-TNF-α (1:1000) were purchased from ABclonal Biotechnology, (Boston, USA). Mouse mAb anti-stat3 (1:1000) and rabbit mAb anti-b-actin (1:1000) were purchased from Cell Signaling Technology (Danvers, USA). Rabbit anti IL-10 monoclonal antibody (1:1000) was purchased from Affbiotech Biosciences (Cincinnati, USA).

The secondary antibodies used for western blot: goat anti-mouse IgG-HRP (1:2000) and goat anti-rabbit IgG-HRP (1:2000) were purchased from Santa Cruz Biotechnology. Goat anti-rat IgG-HRP (1:2000) was purchased from Bioss Inc (Beijing, China). Alexa Fluor 488 goat anti-rabbit IgG (1:2000) was purchased from Invitrogen, and Alexa Fluor 594 goat anti-mouse IgG (1:2000) was purchased from Thermo Fisher Scientific.

The antibody used for IP or Co-IP: rabbit anti-HA tag (4 μg) was purchased from Cell Signaling Technology.

## Results

### CRISPR/CAS9 mediated generation of RH*-Δrop16Δrop18* strain

The CRISPR-Cas9 system was adopted to disrupt the *rop18* gene in the RH-Δ*rop16* strain to generate a double-knockout strain RH-Δ*rop16*Δ*rop18*. A guide RNA (gRNA) and CAS9 expression plasmid pSAG1::CAS9-U6::sgROP18, and the pBlue-5′-3′-ROP18-homo-CAT plasmid containing the CAT reading frame flanking the regions homologous to the *rop18* gene were successfully constructed (Additional file 2: Figure S1a). Tachyzoites of the RH-Δ*rop16* strain were co-transfected with these two plasmids. The recombinant tachyzoites were screened with chloramphenicol, and a monoclonal recombinant was obtained through limiting dilution. Seven single clones were obtained and verified with PCR by confirming the disruption of *rop18* and integration of the CAT marker (Additional file 2: Figure S1b). Numbers 1–3 double-knockout RH-Δ*rop16*Δ*rop18* strain single clones were selected and further confirmed by western blotting (WB) (Additional file 2: Figure S1c).

### Detection of STAT3 activation in RH*-Δrop16* infected HaCaT cells

**Fig. 1:**
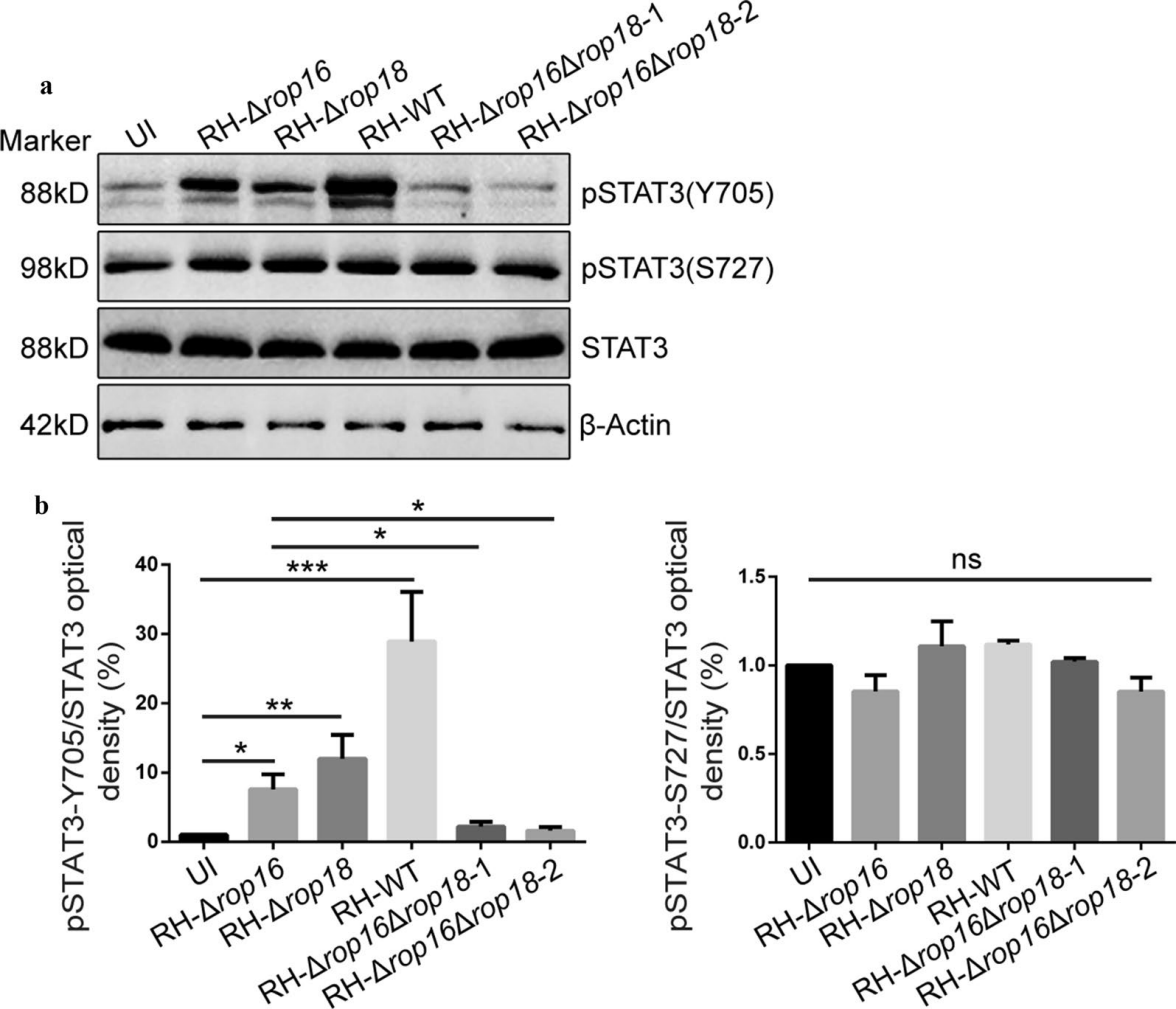
*Tg*ROP18 functions in STAT3 phosphorylation in HaCaT cells during *T. gondii* infection. HaCaT were infected with indicated parasites and harvested. **a** Total proteins of HaCaT cells were loaded and activation of STAT3 was determined by western blot using anti-phospho-Stat3-Y705 and anti-phospho-Stat3-S727. **b** Densitometric quantitation of each band in A was applied using Image J. Statistical analysis was conducted with the Kruskal-Wallis H-test with Bonferroni correction (*P < 0.05, **P < 0.01 and ***P < 0.001). Error bars show the standard errors (SD)

It is reported that ROP16 is the dominant factor activating STAT3 during *T. gondii* infection; a defective STAT3 activation has been observed in the macrophages infected by the RH-*rop16* KO strain [33]. Based on this information, we next tried the other possible parasite kinases on the other cell types. HaCaT cells were infected with RH-WT, RH-Δ*rop16*, RH-Δ*rop18* strains, and two RH-Δ*rop16*Δ*rop18* mono-clones at a MOI of 10 for 30 min, and were subjected to assessment of STAT3 activation (phosphorylation at Y705 and S727). To our surprise, a significant STAT3-Y705 phosphorylation was observed in HaCaT cells when infected with either RH-Δ*rop16* or RH-Δ*rop18* when compared with the uninfected cells or RH-Δ*rop16*Δ*rop18* infected cells, but phosphorylation was still significantly weaker than the RH-WT infected cells (Fig. 1). These results implied that host cell STAT3 could be activated by *Tg*ROP18 in addition to *Tg*ROP16. However, STAT3-S727 phosphorylation which has been reported to facilitate the full activation of STAT3 [37], was not significantly different among all the infected or uninfected groups (Fig. 1).

### STAT3 activation induced by recombinant *TgROP18* added to HaCaT cells culture in a dose-dependent manner

**Fig. 2:**
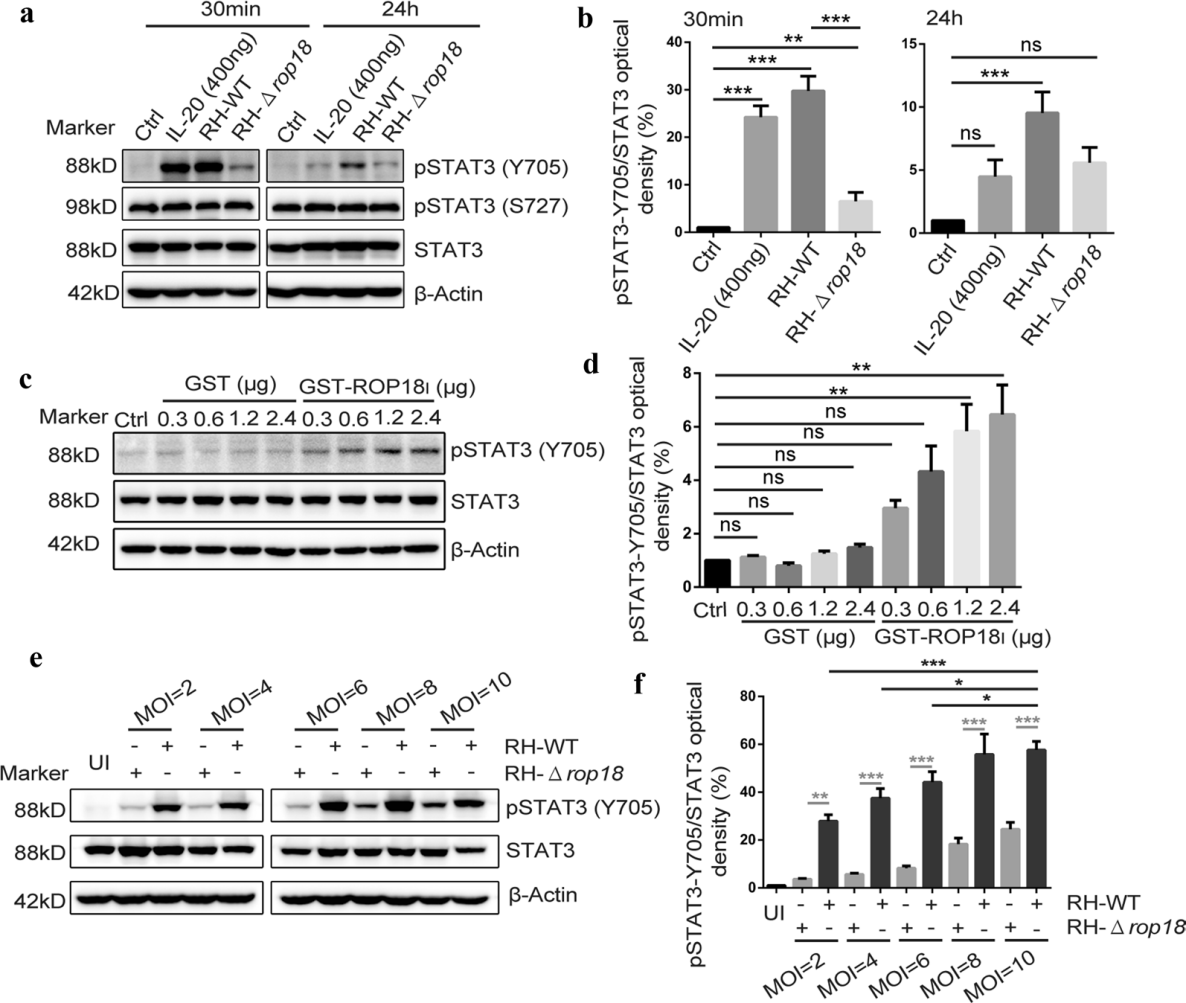
Extracellular *Tg*ROP18 resulted in STAT3-Y705 phosphorylation in HaCaT cells in a dose-dependent manner without affecting the level of total STAT3. **a** HaCaT cells were infected with indicated parasites, the control group was treated with the IL-20 vehicle solution (BSA-PBS), the positive control was stimulated with IL-20. **a**, **c**, **e** The total proteins of HaCaT cells were extracted and subjected to western blot (WB). **b**, **d**, **f** Densitometric quantitation of each band in **a**, **c** and **e** was applied using Image J. **c** HaCaT cells were treated with indicated amount of recombinant GST and GST-ROP18, and elution buffer was used as control. **d** HaCaT cells were infected with gradient MOI of indicated parasite, or uninfected as control. Statistical analysis was conducted by Kruskal-Wallis H-test with Bonferroni correction (*P < 0.05, *P < 0.01 and ***P < 0.001). Error bars show the standard errors (SD)

We further infected HaCaT cells with RH-WT and RH-Δ*rop18* strains for 30 min and 24 h, respectively. Four hundred ng of IL-20 (a ligand of IL20R) was used as a positive control. Then, the STAT3 phosphorylation at Y705 and S727 was detected in the total protein extracted from the different groups of cells. To our surprise, a higher level of STAT3-Y705 phosphorylation was revealed in IL-20 treatment and RH-WT infected groups than in the RH-Δ*rop18* infected group at 30 min post-infection, and no STAT3-Y705 phosphorylation was observed in the control group (untreated cells). Although weak STAT3-Y705 phosphorylation was still observed in these treatment groups at 24 h post-infection, the STAT3-Y705 phosphorylation observed in the RH-WT infected group was stronger than that observed in IL-20 treatment or RH-Δ*rop18* infection group (Fig. 2a, b). Furthermore, we found that *Tg*ROP18 did not affect the expression of total STAT3 and STAT3-S727 phosphorylation (Fig. 2a, b). These results demonstrated that ROP18 significantly phosphorylated STAT3 at Y705 but not at S727 during *T. gondii* infection.

However, previous study elaborated that STAT3 could be activated by overexpressed (intracellular) *Tg*ROP16 but not overexpressed (intracellular) *Tg*ROP18 [33]. Given this fact, we speculated that the phosphorylation of STAT3 was induced by extracellular *Tg*ROP18. Hence, the recombinant GST-ROP18 expressed by *E. coli* was added to the cell culture medium to determine the effect of *Tg*ROP18 on STAT3 activation. A co-localization of IL20RB and GST-ROP18 on the HaCaT cell membrane, but no co-localization between IL20RB and GST, was observed (Additional file 3: Figure S2). Meanwhile, the phosphorylation of STAT3 was detected in HaCaT cells which had been incubated with different concentrations of recombinant GST-ROP18 for 30 min. Intriguingly, we found that the STAT3-Y705 phosphorylation was distinctly induced by GST-*Tg*ROP18 in a dose-dependent manner, but the STAT3 expression was not affected by this recombinant protein added to the cell culture (Fig. 2a, d). This result suggested that the STAT3 activation could be mediated by extracellular *Tg*ROP18 without changing the total STAT3 level. To further confirm this phenomenon, HaCaT cells were infected with RH-WT and RH-Δ*rop18* tachyzoites at a MOI of 2, 4, 6, 8 and 10 for 30 min, separately. We found that RH-WT induced a much higher level of STAT3-Y705 phosphorylation than RH-Δ*rop18* at the same MOI index, and the phosphorylation levels resulted by both of these two strains increased with the MOI index from 2–8 (at MOI 2, 4, 6 and 8), before stabilizing between MOI 8 and 10 (Fig. 2e, f).

### *Tg*ROP18 interacts with host cell IL20RB at the extracellular domain CFPN1-ROP18

**Fig. 3:**
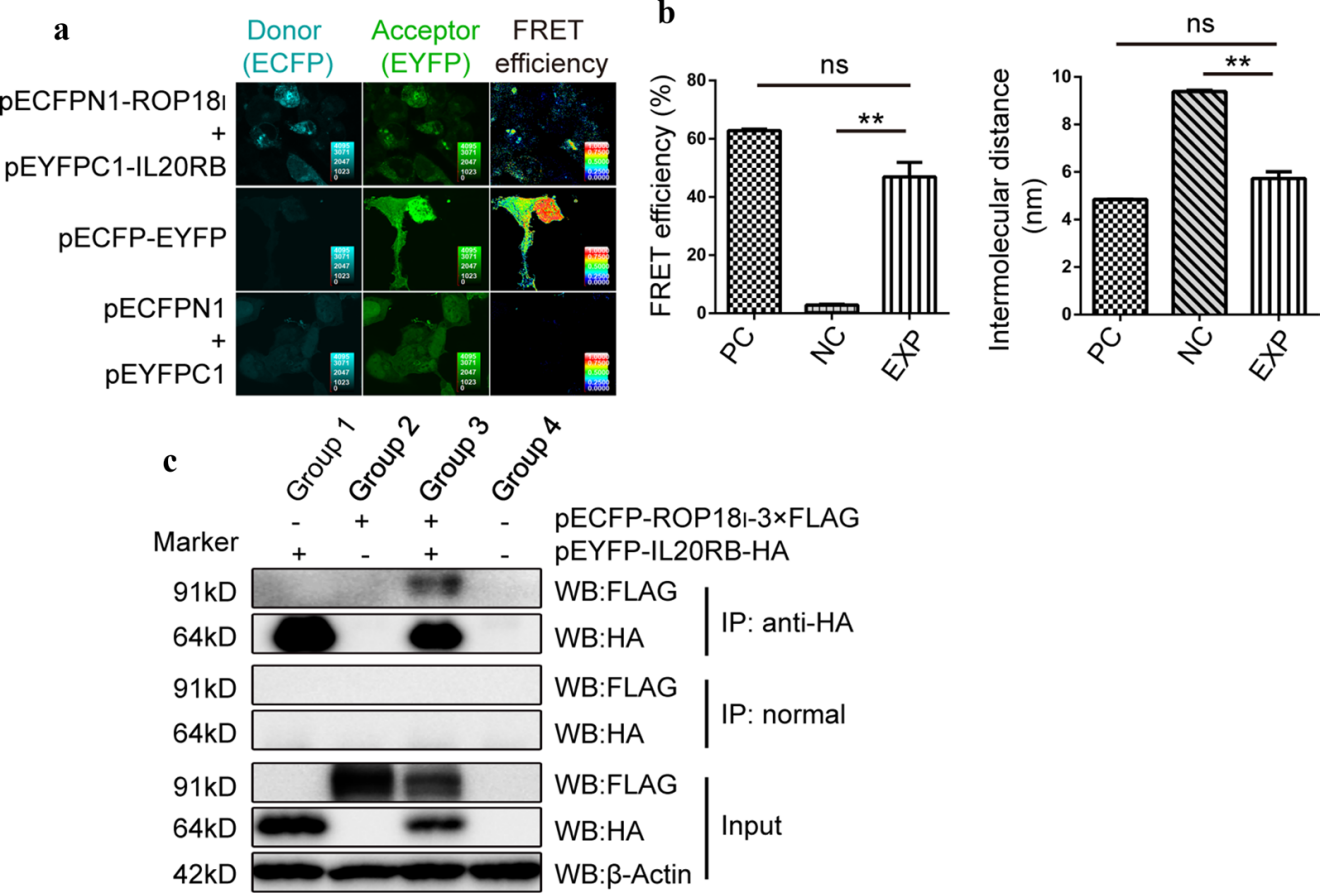
Identification of IL-20RB interacting with *Tg*ROP18 with FRET and Co-IP. **a** Identification of the interaction using FRET. The COS-7 cells co-transfected with pECFPN1-ROP18 and pEYFPC1-IL20RB (EXP) were used to detect the FRET efficiency between CFPN1-ROP18 and YFPC1-IL20RB. The COS-7 cells co-transfected with pECFPN1 and pEYFPC1 were used for negative control (NC) and the cells transfected with pECFPN1-EYFP as the positive control (PC). The FRET efficiency is shown in the right column where a color matched FRET efficiency scale is indicated for each image. The red end of the scale indicates a higher FRET efficiency. **b** The calculated FRET efficiency (left) and intermolecular distance (right) between ROP18 and IL20RB is shown as a column. **c** The interaction of ROP18 with IL20RB was further confirmed through immunoprecipitation assay (Co-IP) *in vitro*. Group 1: cells transfected with pEYFP-IL20RB-HA; Group 2: cells transfected with pCFP-ROP18_I_-3×FLAG; Group 3: cells co-transfected with pCFP-ROP18_I_-3×FLAG and pEYFP-IL20RB-HA; Group 4: negative control group

**Fig. 4:**
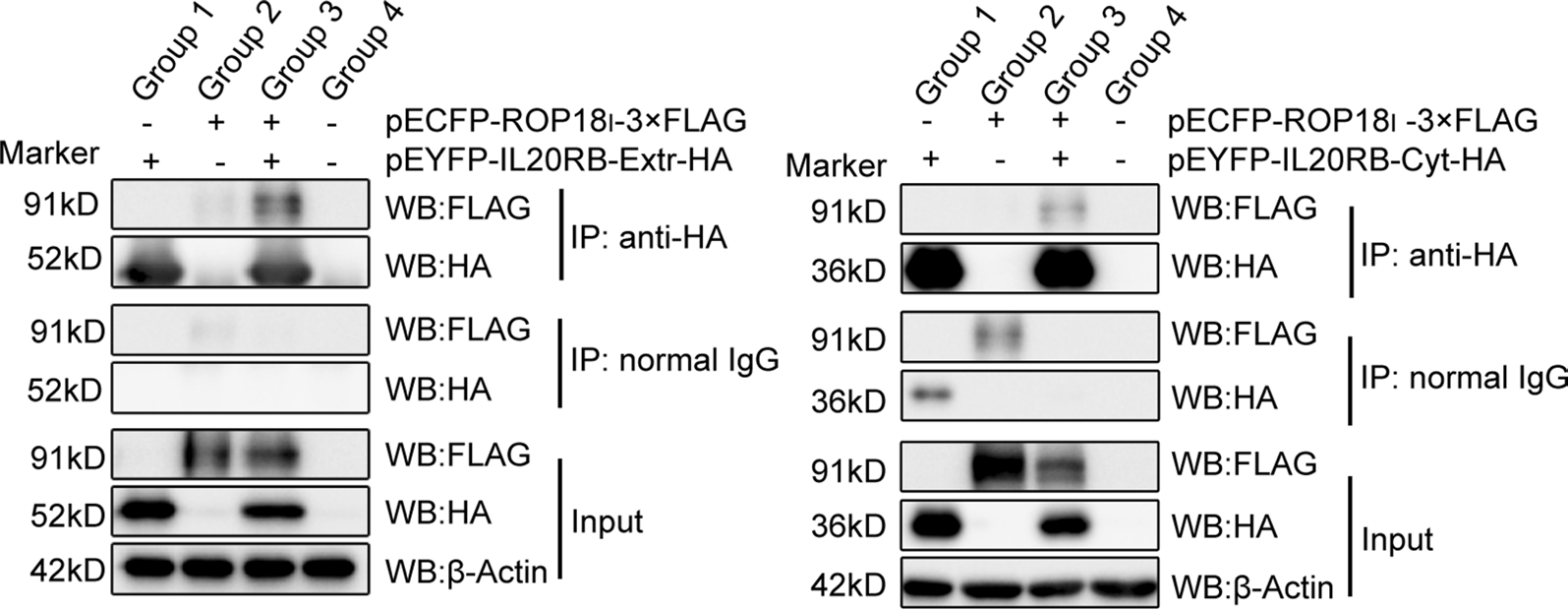
Identification of IL-20RB extracellular domain as the *Tg*ROP18-binding domain. The interaction of ROP18 with the Extr/Cyt domain of IL20RB was identified with Co-IP. Lysates of COS-7 cells transiently transfected or co-transfected with the indicated plasmids, were immunoprecipitated with anti-HA. Starting fractions (Input) and immunoprecipitates (IP) were analyzed with WB to detect FLAG-ROP18 and the HA-Extr/Cyt domain of IL20RB. Group 1: cells transfected with pEYFPC1-IL20RB-Extr-HA or pEYFPC1-IL20RB-Cyt-HA; Group 2: cells transfected with pCFP-ROP18_I_-3×FLAG; Group 3: cells co-transfected with pCFP-ROP18_I_-3×FLAG and pEYFPC1-IL20RB-Extr-HA or pEYFPC1-IL20RB-Cyt-HA; Group 4: negative control group

In our previous study, a bi-molecular fluorescence complementation (BiFc) protein interaction screening was performed to identify the human interactom of *Tg*ROP18, and the human IL20RB was screened as an interaction protein of *Tg*ROP18 [35]. Fluorescence resonance energy transfer (FRET) was conducted to further confirm this interaction. The results indicated that the FRET efficiency and intermolecular distance of CFP-*Tg*ROP18 and IL20RB-YFP (in the pCFPN1-ROP18/pYFPC1-IL20RB co-transfection group) was not significantly different from that of CFP-YFP (in the positive control group), but was significantly different from that of CFP and YFP (in the negative control group) (Fig. 3a, b). This interaction was also verified by Co-IP assay *in vitro*. FLAG tagged ROP18 and HA tagged IL20RB were detected in the immunoprecipitates with anti-HA antibody, but not in those precipitated with normal IgG (Fig. 3c). Because IL20RB is known as a transmembrane glycoprotein, to further investigate which domain of IL20RB is binding with *Tg*ROP18, the Co-IP assay was performed to detect the interaction of *Tg*ROP18 with the extracellular/intracellular domain of IL20RB. The WB results demonstrated that *Tg*ROP18 could interact with the extracellular domain of IL20RB, but not its intracellular (Cyt) domain (Fig. 4).

All these data indicated that host cell IL20R could be a receptor of *Tg*ROP18 to activate STAT3 through targeting the extracellular domain of its beta chain IL20RB.

### STAT3 activated by ROP18 in cells expressing IL20RB

**Fig. 5:**
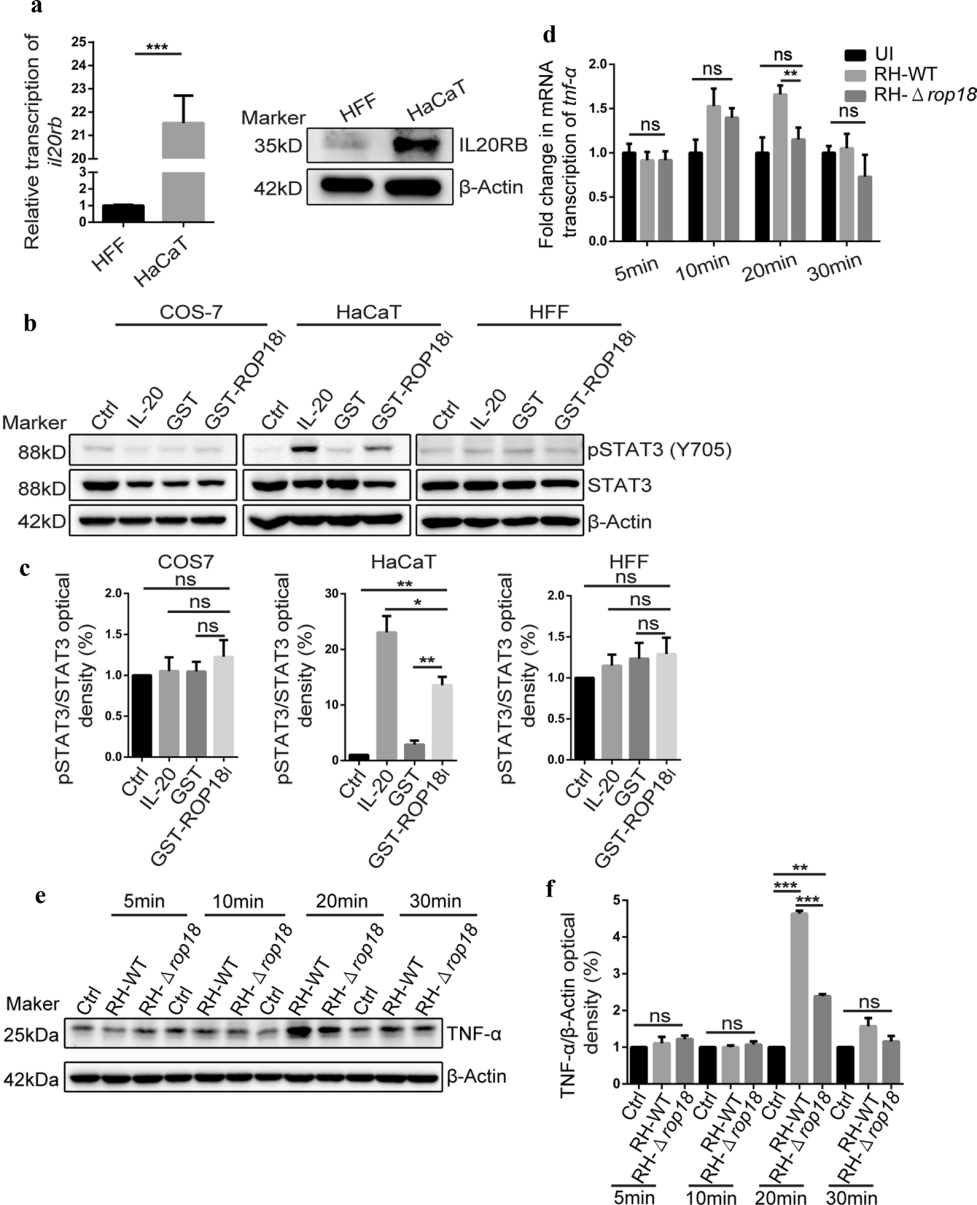
Detection of the activation of IL20R-JAK/STAT3 pathway in HaCaT cells treated with recombinant *Tg*ROP18 protein. **a** Transcription and expression of IL20RB in HaCaT relative to HFF was detected by qRT-PCR and Western blot. **b** COS-7, HaCaT, and HFF cells were treated with IL-20, recombinant GST, or recombinant GST-ROP18 for 30 min, and elution buffer treated cells were used as negative control. STAT3-pY705 level was determined by WB. **c**, **f** Densitometric quantitation of each band in B or E was applied using Image J. **d**, **e** HaCaT cells were infected with indicated parasites for the indicated times. TNF-α transcription and translation was detected by qRT-PCR and WB. Statistical analysis was performed with the Kruskal-Wallis H-test with Bonferroni correction (**P < 0.01)

**Fig. 6:**
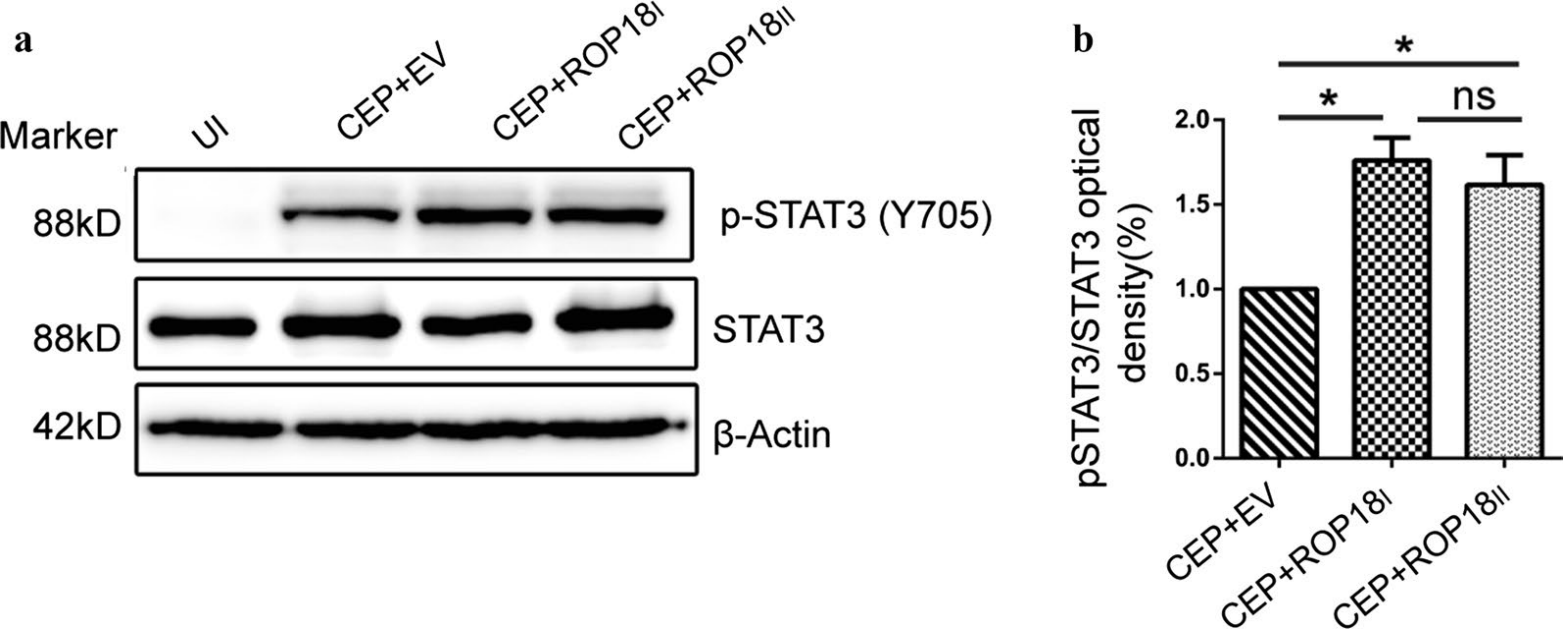
Both *Tg*ROP18 and *Tg*ROP18 induce STAT3 activation in HaCaT cells. **a** HaCaT were infected with indicated parasites for 30 min. Cell lysates were examined for STAT3 activation by WB. **b** Densitometric quantitation of STAT3 phosphorylation was applied with Image J and normalized to the total STAT3. Statistical analysis was conducted with the Kruskal-Wallis H-test with Bonferroni correction (*P < 0.05), and error bars show the standard errors of the mean for three independent experiments

Since *Tg*ROP18-IL20RB interaction occurred in the extracellular domain of IL20RB, we speculated that the IL20RB receptor on the host cell membrane was responsible for the STAT3 activation induced by ROP18, thus, the expression of IL20RB in several types of cells was examined. The mRNAs of IL20RA and IL22RA1, which comprise the alpha chain of IL20R and are required for complete IL20R formation and indispensable for inducing downstream signaling events, were also detected. Among COS-7, HEK293T, HFF and HaCaT cells, IL20RB was found to be transcribed in higher levels in HaCaT cells, as well as the other components to form the IL20R receptor (Additional file 4: Figure S3). We further detected the IL20RB protein in HFF and HaCaT cells with WB, and found IL20RB was highly expressed in HaCaT cells, instead of HFF cells (Fig. 5a). Considering IL20RB is a transmembrane glycoprotein, and the binding site with *Tg*ROP18 is the extracellular domain, it possibly mediated the signal transduction of *Tg*ROP18 during invasion when the parasite or the secreted *Tg*ROP18 contacted the cell membrane. To test this, COS-7, HFF and HaCaT cells were selected and treated with recombinant GST-ROP18 for 30 min, with the cells treated with IL-20 as a positive control, and the untreated cells and the cells treated with recombinant GST as negative controls. Our results indicated that STAT3-Y705 phosphorylation was significantly induced by the recombinant ROP18 and IL-20 in HaCaT cells, but not in COS-7 and HFF cells (Fig. 5b, c). Therefore, we concluded that *Tg*ROP18 mediated STAT3 activation in the cells expressing a high level of IL20RB such as HaCaT cells. We further examined the transcription and expression of *tnf-α* and *iNOS*, a downstream gene of the IL20R-JAK/STAT3 pathway regulated by STAT3 activation, using HaCaT cells uninfected or infected with RH-WT or RH-Δ*rop18.* The most significant *tnf-α* and *iNOS* transcription; and *tnf-α* translation was observed at 20 min post-infection in both RH-WT and RH-Δ*rop18* infection groups, but RH-WT resulted in a significantly higher level than RH-Δ*rop18* (Fig. 5d-f, Additional file 5: Figure S4a). As STAT3 is the main transcription factor activated by IL-20 receptors and can be activated by IL10 family members, such as IL-10, IL-20, and IL-24, in HaCaT cells [30, 31], we further confirmed that these IL10 family members were not produced by HaCat cells after *T. gondii* infection (Additional file 5: Figure S4b-e). However, a significant STAT3 activation in HaCaT cells, by the recombinant ROP18 and endogenous ROP18 secreted by *T. gondii* was observed in our study (Figs. 1, 2, 5, 6).

Meanwhile, using qRT-PCR, we also detected the transcription of *il20rb* and one of its alpha chain *il20ra*1 in HaCaT cells after infection by RH-WT and RH-Δ*rop18* at 5, 10, 20, and 30 min post-infection. Using WB, the translation of *il20rb* in HaCaT cells after treatment with 400 ng of IL-20 and infection by RH-WT or RH-Δ*rop18* was detected at 30 min post-infection. Our results indicated that both the transcription and translation of IL20RB was not affected by *Tg*ROP18 until after 30 min post-infection (Additional file 6: Figure S5).

### Both type I and type II *Tg*ROP18 activate host cell STAT3

To explore whether type II ROP18 (*Tg*ROP18II) can activate host cell STAT3 in the same way as *Tg*ROP18I, HaCaT cells were infected with the type III strain CEP, and the CEP expressing type I ROP18 (CEP-*Tg*ROP18I); and type II ROP18 (CEP-*Tg*ROP18II), for 30 min. The cells were harvested, and total protein was extracted followed by detection of STAT3-Y705 phosphorylation. Compared to the CEP (type III) strain, both CEP-*Tg*ROP18I, and CEP-*Tg*ROP18II infection showed significant STAT3 activation in HaCaT cells, but no significant difference existed between CEP-*Tg*ROP18I, and CEP-*Tg*ROP18II infection (Fig. 6 This result indicated that both type I and type II ROP18 could similarly activate STAT3 in HaCaT cells.

## Discussion

**Fig. 7:**
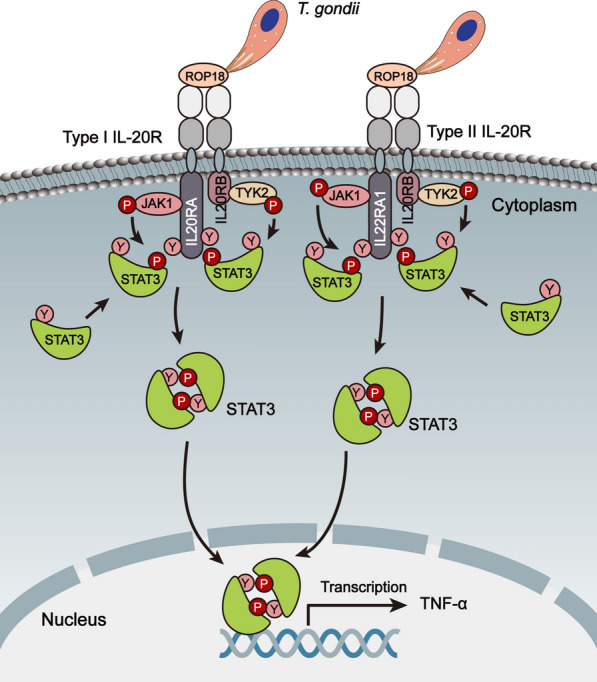
Schematic illustration of the JAK/STAT3 pathway activated by *Tg*ROP18 through targeting IL20RB. Type I IL-20R is shown on the left and type II IL-20R on the right. *Tg*ROP18 is secreted from rhoptries of *T. gondii* and binds to host cell IL20RB on the extracellular domain during invasion. The β-chain of IL20R (IL20RB) associates with and phosphorylates TYK2, while the α-chain (IL20RA or IL22RA1) associates with and phosphorylates JAK1. Activated JAK1 and TYK2 undergo cross-phosphorylation and autophosphorylation. Thereafter, the tyrosine residues within the intracellular domain of IL-20RA (IL-20RA1) are phosphorylated and create the docking sites for STAT3. Meanwhile, free STAT3 in the cytoplasm are recruited and phosphorylated by JAK1 and TYK2. Phosphorylated STAT3 then forms homodimers and translocates to the nucleus to initiate transcription of STAT3 responsive genes such as TNF-α through STAT-responsive promoter elements. *Abbreviations*: STAT3, signal transducers and activators of transcription 3; JAK1, Janus Kinase 1; TYK2, Tyrosine Kinase 2; Y, tyrosine residue; P, phosphorylation

In this study, we demonstrated that host cell STAT3 could be activated by *T. gondii* virulence factor ROP18 (of both type I and type II stains) to regulate host innate immune responses in HaCaT cells during invasion. The recombinant *Tg*ROP18 and the native *Tg*ROP18 secreted by parasites bound to IL20RB on the cell membrane to activate STAT3 in HaCaT cells*. Tg*ROP18 interacted with host IL20RB and the binding site was on the IL20RB extracellular domain. In addition, *Tg*ROP18 only mediated STAT3 activation in the cells expressing IL20R with a high level (such as HaCaT cells), and ultimately resulted in a promotion of TNF-α and iNOS transcription which represented the activation of STAT3 (Fig. 7, Additional file 5: Figure S4a).

We therefore deduced the whole process as follows. Upon *T. gondii* infection, it first attaches firmly to the cell membrane. After attaching, the host cell manipulation by *T. gondii* initiates for less than a minute [38]. During this period, *Tg*ROP18 secreted from *T. gondii* rhoptries bind to the host cell IL20RB, which was only expressed in some cell types on the membrane, to induce downstream signaling events. The interaction of ROP18 and IL20RB was further confirmed by FRET and Co-IP. It has been reported that overexpressed ROP18 had no effect on STAT3 activation [19, 33]. Therefore, *Tg*ROP18 may function through binding with IL20RB on the cell membrane to activate STAT3 and JAK. This phenomenon was observed in HaCaT cells with high IL20RB expression, but not in COS-7 and HFF cells with very low IL20RB expression. HaCaT expressed both type I and type II IL-20 receptors with high levels and showed significant STAT3 activation when treated with recombinant GST-ROP18.

It is generally believed that JAK/STAT3 pathway activation is typically presented with STAT3-Y705 phosphorylation, the phosphorylated STAT3 then form homodimers and translocate to the cell nucleus where they induce transcription of target genes such as TNF-α and iNOSα [39]. Therefore, STAT3-Y705 phosphorylation was used to evaluate the activation of the JAK/STAT3 pathway. Though the host cell STAT3 activation by overexpressed ROP16 but not overexpressed ROP18 is well clarified [33], we still observed that the *Tg*ROP16 defective RH strain showed a significant but relatively lower STAT3 activation compared to the wild type RH strain. Combined with the finding that GST-ROP18 incubated cells showed significant STAT3 activation compared to the GST incubated cells, we can deduce that STAT3 activation by *Tg*ROP18 was due to the binding of *Tg*ROP18 on the extracellular domain of IL20RB. These results also explained why the STAT3 activation mediated by *Tg*ROP18 was considerably more distinct in HaCaT cells at 20–30 min post-infection, earlier than the point at which most tachyzoites were attaching to, but not yet entering the cells. Though it has been reported that IL20RB can induce not only STAT3, but also STAT1 and STAT5, STAT1 and STAT5 activation needs a much higher concentration of IL-19, IL-20, or IL-24 than STAT3 does [20, 31–34]. Moreover, STAT3 is the main transcription factor activated by type I and type II IL-20 receptors and has been verified to be activated by IL-19, IL-20, and IL-24 in HaCaT cells [30, 31].

Therefore, we further confirmed that these IL10 family members were not produced by HaCat cells after *T. gondii* infection (Additional file 5: Figure S4b-e). However, a significant activation effect of ROP18 on STAT3 through the IL20RB during *T. gondii* infection in HaCaT cells was observed in our study. On the other hand, examination of whether STAT1 and STAT5 signaling can be induced by ROP18 through IL20R receptor complex, and its potential functions, requires further research.

In this study, we could not exclude the possibility of IL19, IL-20 or IL-24 binding with IL-20R other than ROP18 *in vivo*. Considering the interaction of *Tg*ROP18 with IL20RB occurred at an early infection stage, and the major cytokines strongly induced by *T. gondii* at this stage are type I cytokines, and not IL-20 subfamily cytokines [22, 40–43], therefore, the existence of a competition between ROP18 and the IL-20 subfamily working on IL-20R is not possible. It is possible that *Tg*ROP18 functions on IL-20R at the early stage of infection, while IL-20 subfamily cytokines work on IL-20R at the relatively later stage of infection.

The activation of the JAK/STAT3 pathway enhances the expression of its downstream proinflammatory factors such as TNF-α, which are closely related to early inflammatory initiation and immune responses. In a previous study, TNF-α was found to be upregulated in *T. gondii* infection [44]. Furthermore, TNF-α is not only an activator inducing higher levels of IFN-γ [44] but also an important activator of the NF-κB pathway [45]; both play an important role in anti-*T. gondii* infection. In previous studies, ROP18 mainly exerts their immunosuppressive action in *T. gondii* infection to promote their survival [13, 25, 27, 46]. In our study, we revealed that *Tg*ROP18 activated the IL20R-JAK/STAT3 pathway for anti-infection against *T. gondii*, and mainly played a role in early infection. Once the invasion is accomplished, other signaling pathways play important roles in anti-*T. gondii* infection and will be soon triggered by ROP18, such as NF-κB signaling [25, 47]. The recent research has clarified that *T. gondii* inhibits host immunity through ROP16-mediated activation of STAT3 [48], which may explain why the *Tg*ROP18 activated IL20R-JAK/STAT3 pathway only caused transiently enhanced production of TNF-α during early infection, since the anti-infection effect was covered by ROP16.

Ultimately, similar STAT3 activation results were observed in CEP-*Tg*ROP18I and CEP-*Tg*ROP18II infected HaCaT cells at 30 min post-infection, which suggested that both type I and type II ROP18 could activate STAT3 in HaCaT cells at the early stage of infection. However, whether they were involved in the same mechanism still needs to be confirmed.

## Conclusions

This study determined that the immune-related receptor protein IL20RB was targeted by *Tg*ROP18 on its extracellular domain upon *T. gondii* invasion, and led to synergic activation of the host JAK/STAT3 pathway together with *Tg*ROP16, triggering proinflammatory immune responses against *T. gondii*. Herein, we elucidated a novel molecular mechanism of manipulation of *Tg*ROP18 on host immune responses and provided new insights into the *T. gondii*-host interaction.

## Supplementary information

**Additional file 1: Table S1.** Primers used in this study.

**Additional file 2: Figure S1.** Gene knockout of *rop18* from the RH-*Δrop16* strain. **a** Schematic of the CRISPR/CAS9 strategy disrupting the *rop18* locus by insertion of a CAT marker in the genome of RH-*Δrop16*. **b** PCR verification of *rop18* disruption in seven colonies, compared with the RH-*Δrop16* strain. **c** WB confirmation of ROP18 expression silence in three colonies.

**Additional file 3: Figure S2.** Detection of the co-localization of ROP18 and IL20RB on the HaCaT cell membrane. HaCaT cells were incubated with 1 mg of GST or GST-ROP18 separately. The cells were fixed and probed with mouse anti-GST and rabbit anti IL20RB antibodies, then incubated with the fluorescence secondary antibodies. The results showed a co-localization of IL20RB and GST-ROP18 on the HaCaT cell membrane, but no co-localization was observed between IL20RB and GST.

**Additional file 4: Figure S3.** High transcription levels of IL20R subunits identification in HaCaT cells. Endogenous transcription levels of IL20RA, IL22RA1 and IL20RB in COS-7, HEK293T, HFF and HaCaT cells were detected with RT-PCR.

**Additional file 5: Figure S4.** Comparison of the transcription and translation levels of iNOS, IL20, IL22 and IL10 in RH-WT or RH-∆*rop18* infected HaCaT cells, and the uninfected cells. **a** iNOS transcription level in the RH infection group was significantly higher than in the other groups at 20 min post-infection, but not at 10, and 30 min (*P < 0.05). **b**-**d** No significant difference was found in the IL20, IL22, and IL10 transcription level, and the translation level of IL10 at both 30 min and 24 h post-infection among the indicated groups.

**Additional file 6: Figure S5.***Tg*ROP18 did not regulate the transcription and expression of IL20RB and IL22RA1. HaCaT cells were infected with indicated parasites for the indicated time. The transcriptional levels of IL20RB (**a**) and IL22RA1 (**c**) in HaCaT cells were detected with qRT-PCR. **b** The expression of IL20RB was detected by WB, no significant difference was found among the indicated groups (*P < 0.05).

## Data Availability

Not applicable.

## Abbreviations

ROPs: rhoptry proteins
GRAs: dense granule proteins
MICs: microneme proteins
JAK: Janus kinase
STAT3: signal transducers and activators of transcription 3
*Tg*ROP18: *Toxoplasma gondii* rhoptry protein 18
*Tg*ROP16: *Toxoplasma gondii* rhoptry protein 16
HaCaT: human keratinocytes cells
HFF: human foreskin fibroblasts
COS-7: African green monkey kidney cells transformed by SV40
HEK 293T: human embryonic kidney 293T
IL20R: interleukin 20 receptor
IL20RB: interleukin 20 receptor subunit b
Co-IP: co-immunoprecipitation
WB: western blot
PCR: polymerase chain reaction
RT-PCR: reverse transcription polymerase chain reaction
FRET: fluorescence resonance energy transfer
