# Supplementary material for: TgROP18 targets IL20RB for host-defense-related-STAT3 activation during Toxoplasma gondii infection
Source: Parasit Vectors. 2020 Aug 7;13:400. doi: 10.1186/s13071-020-04251-7 (PMC7412674; doi:10.1186/s13071-020-04251-7)
Supplement: Supplementary file 1 — Additional file 1: Table S1. Primers used in this study. [file 13071_2020_4251_MOESM1_ESM.docx]

**Additional file 1: Table S1.** Primers used in this study

| Primers | Sequence | Used for |  |
| --- | --- | --- | --- |
| IL20RB-F | GTCGACATGCAGACTTTCACAATGGTTC | To amplify fragment of *il20rb* for pBlunt-IL20RB-HA and pEYFPC1- IL20RB-HA cloning |  |
| IL20RB-HA-R | CCGCGGTCAGGCATAATCGGGTACATCGTAAGGGTATGAGATCCAGGCCCTGAG |  |  |
| ROP18-F | ACGCGTCGACATGTTTTCGGTACAGCGGCC | To amplify fragment of *rop18* for pCFPN1-ROP18-3×FLAG cloning |  |
| ROP18-3×flag-R | TCCCCGCGGTTACTTATCGTCATCGTCTTTGTAATCAATATCATGATCCTTGTAGTCTCCGTCGTGGTCCTTATAGTCTTCTGTGTGGAGATGTTCCTGC |  |  |
| IL20RB-Extr-F | GTCGACGATGAAGTGGCCATTCTG | To amplify fragment of *il20rb* for pBlunt-IL20RB-Extr-HA and pEYFPC1- IL20RB-Extr-HA cloning |  |
| IL20RB-Extr-HA-R | CCGCGGTCAGGCATAATCGGGTACATCGTAAGGGTACAGGGGAATGGCCT |  |  |
| IL20RB-Cyt-F | GTCGACTGGAAAATGGGCCGGCTGCT | To amplify fragment of *il20rb* for pBlunt-IL20RB-Cyt-HA and pEYFPC1- IL20RB-Cyt-HA cloning |  |
| IL20RB-Cyt-HA-R | CCGCGGTCAGGCATAATCGGGTACATCGTAAGGGTATGAGATCCAGGCCCTGA |  |  |
| IL20RB-RT-F | GGGGAGTACGAGAGCCTGTA | *il20rb* amplification for RT-PCR |  |
| IL20RB-RT-R | CTTCACGAATGTCTGGGCCT |  |  |
| IL22RA1-RT-F | TGCAGCACACTACCCTCAAG | *il22ra1* amplification for RT-PCR |  |
| IL22RA1-RT-R | GACGTTCAGGGAGTTGGGAG |  |  |
| IL20RA-RT-F | GCAGAACAATACCCCCGGAT | *il20ra* amplification for RT-PCR |  |
| IL20RA-RT-R | TTTTCTCCTGGTGGCCTGTC |  |  |
| IL20RB-q-F | CATGTTTGCTCACAGATGAAGT | *il20rb* amplification for qRT-PCR |  |
| IL20RB-q-R | ATCACTGGGCTCCACATCAA |  |  |
| IL22RA1-q-F | CTCTGCAGCACACTACCCTC | *il22ra1* amplification for qRT-PCR |  |
| IL22RA1-q-R | ATGTCTTCCAGGGTTAGCCG |  |  |
| TNFα-q-F | GTGCTTGTTCCTCAGCCTCT | *tnf-α* amplification for qRT-PCR |  |
| TNFα-q-R | ATGGGCTACAGGCTTGTCAC |  |  |
| INOS-F | GTCAGAGTCACCATCCTCTTTG | *inos* amplification for qRT-PCR |  |
| INOS-R | GCAGCTCAGCCTGTACTTATC |  |  |
| IL10-F | GACTTTAAGGGTTACCTGGGTTG | *il10* amplification for qRT-PCR |  |
| IL10-R | TCACATGCGCCTTGATGTCTG |  |  |
| IL20-F | TTTTCTGAGATACGGGGCAGT | *il20* amplification for qRT-PCR |  |
| IL20-R | GTCTTAGCAAATGGCGCAGGA |  |  |
| IL19-F | GGCTCCTGGGTACAATACTGA | *il19* amplification for qRT-PCR |  |
| IL19-R | GGTGTCCTTAGCTTGGATGGC |  |  |
| IL22-F | GCTTGACAAGTCCAACTTCCA | *il22* amplification for qRT-PCR |  |
| IL22-R | GCTCACTCATACTGACTCCGT |  |  |
| GAPDH-F | GGAGCGAGATCCCTCCAAAAT | *gapdh* amplification for RT-PCR and qRT-PCR |  |
| GAPDH-R | GGCTGTTGTCATACTTCTCATGG |  |  |
| ROP18-GST-F | CGGGATCCATGGAAAGGGCTCAACACC | To amplify fragment of *rop18* for pGEX-4T-2-ROP18 cloning and GST-ROP18 purification |  |
| ROP18-GST-R | ACGCGTCGACTTAGTGATGATGATGATGATGCTTGTCATCGTCATCCTTG |  |  |
| GST-6×His-F | ATCTGGTTCCGCGTGGATCCCATCATCATCATCATCACTAAGTCGACTCGAGCGGCCG | To insert 6×His into pGEX-4T-2 for the purification of GST |  |
| GST-6×His-R | CGGCCGCTCGAGTCGACTTAGTGATGATGATGATGATGGGATCCACGCGGAACCAGAT |  |  |
| Q5-ROP16 -F: | TGCACGATACATGTCGTTTGGTTTTAGAGCTAGAAATAGC | To replace the CRISPR plasmid target sequence of guideRNA with the target sequence of *rop* 16 |  |
| Q5-ROP16 -R | AACTTGACATCCCCATTTAC |  |  |
| SgRNA-1 | TGCACGATACATGTCGTTTG | Introduce the DHFR drug screen cassette (3600bp) into 65th base of the coding sequence for the knockout of *rop16* |  |
| 5'-homo -ROP16-F | CCCAAGCTTACTGGGGCAGCCGTCACG | To amply fragment of 5’-homo for the knockout of *rop*16 |  |
| 5'-homo -ROP16-R | CGGATATCCTTGCGACAAACAAGATCAC |  |  |
| 3'-homo -ROP16-F | CGGGATCCGGTGTAAGGTTCCCACC | To amply fragment of 3’-homo for the knockout of *rop16* |  |
| 3'-homo -ROP16-R | GCACTAGTCATTGGCCTAAACTTG |  |  |
| DHFR-F | CGGATATCAAGCTTCGCCAGGCTGTAAATC | To amply fragment of DHFR for the knockout of *rop16* |  |
| DHFR-R | CGGGATCCCAGGAATTCATCCTGCAAGTGC |  |  |
| 5'-homo -ROP18-F | CTCGAGGTCGACGGTATCGATAAGCTTGCAGTTGCACAGGGACGACG | To amply fragment of 5’-homo for the knockout of *rop18* |  |
| 5'-homo -ROP18-R | CACTAGTTCTAGAGCGGCCTCGAGAGGTGGCCGCTGTACCG |  |  |
| 3'-homo -ROP18-F | ATCAAGCTGGGTTTAGCGACTCTTCTCCC | To amply fragment of 3’-homo for the knockout of *rop18* |  |
| 3'-homo -ROP18-R | GGCGGCCGCTCTAGAACTAGTGGATCCGGATGCTGGCTGTCCCTCTAA |  |  |
| CAT-F | TCGAGGCCGCTCTAGAACTAGTG | To amply fragment of CAT drug screen cassette for the knockout of *rop18* |  |
| CAT-R | GGGAGAAGAGTCGCTAAACCCAGCTTGATATCGAATTCCTGCAGC |  |  |
| PCR1-F | TGTGTGTGTACATGCACGCTTGT | PCR1 |  |
| PCR1-R | CGTTGTGCTCACTTCTCAAATC |  |  |
| PCR2-F | ATCAGCAGTGACTACAGCTT | PCR2 |  |
| PCR2-R | GGATGCTGGCTGTCCCTCTAACATCACAA |  |  |
| PCR3-F | TTCCAGTTTCGCTTTGAGT | PCR3 |  |
| PCR3-R | TAACGATTTC GCTCTACGCC |  |  |
| SgRNA-2 | TACGCGTACCGTCGTCCGAA | Introduce the CAT drug screen cassette (1398bp) into 46th base of the coding sequence for the knockout of *rop18* |  |
