# Supplementary figures and images for: TgROP18 targets IL20RB for host-defense-related-STAT3 activation during Toxoplasma gondii infection
Source: Parasit Vectors. 2020 Aug 7;13:400. doi: 10.1186/s13071-020-04251-7 (PMC7412674; doi:10.1186/s13071-020-04251-7)

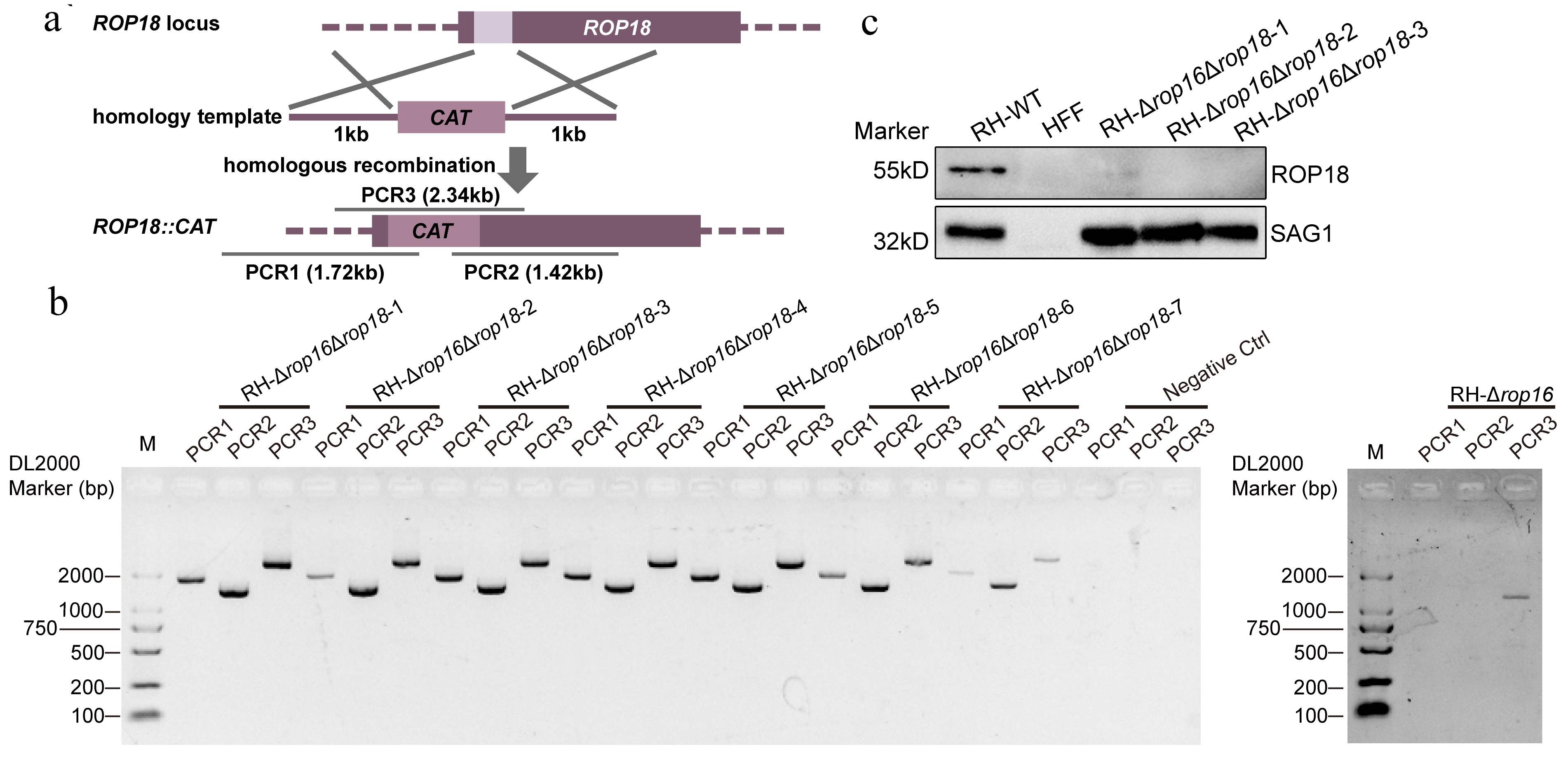

Supplement: Supplementary file 2 — Additional file 2: Figure S1. Gene knockout of rop18 from the RH-Δrop16 strain. a Schematic of the CRISPR/CAS9 strategy disrupting the rop18 locus by insertion of a CAT marker in the genome of RH-Δrop16. b PCR verification of rop18 disruption in seven colonies, compared with the RH-Δrop16 strain. c WB confirmation of ROP18 expression silence in three colonies. [file 13071_2020_4251_MOESM2_ESM.tif]

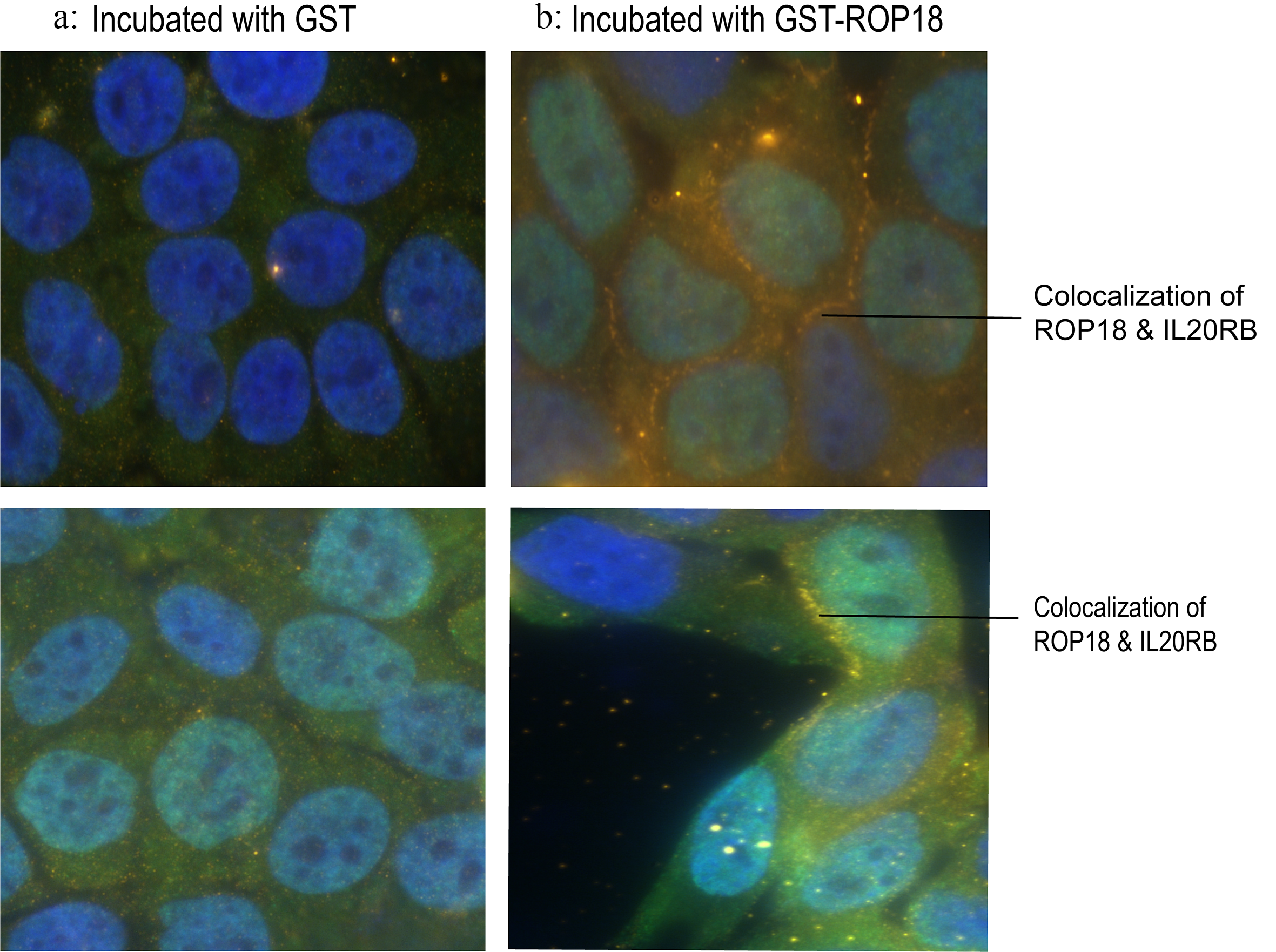

Supplement: Supplementary file 3 — Additional file 3: Figure S2. Detection of the co-localization of ROP18 and IL20RB on the HaCaT cell membrane. HaCaT cells were incubated with 1 mg of GST or GST-ROP18 separately. The cells were fixed and probed with mouse anti-GST and rabbit anti IL20RB antibodies, then incubated with the fluorescence secondary antibodies. The results showed a co-localization of IL20RB and GST-ROP18 on the HaCaT cell membrane, but no co-localization was observed between IL20RB and GST. [file 13071_2020_4251_MOESM3_ESM.tif]

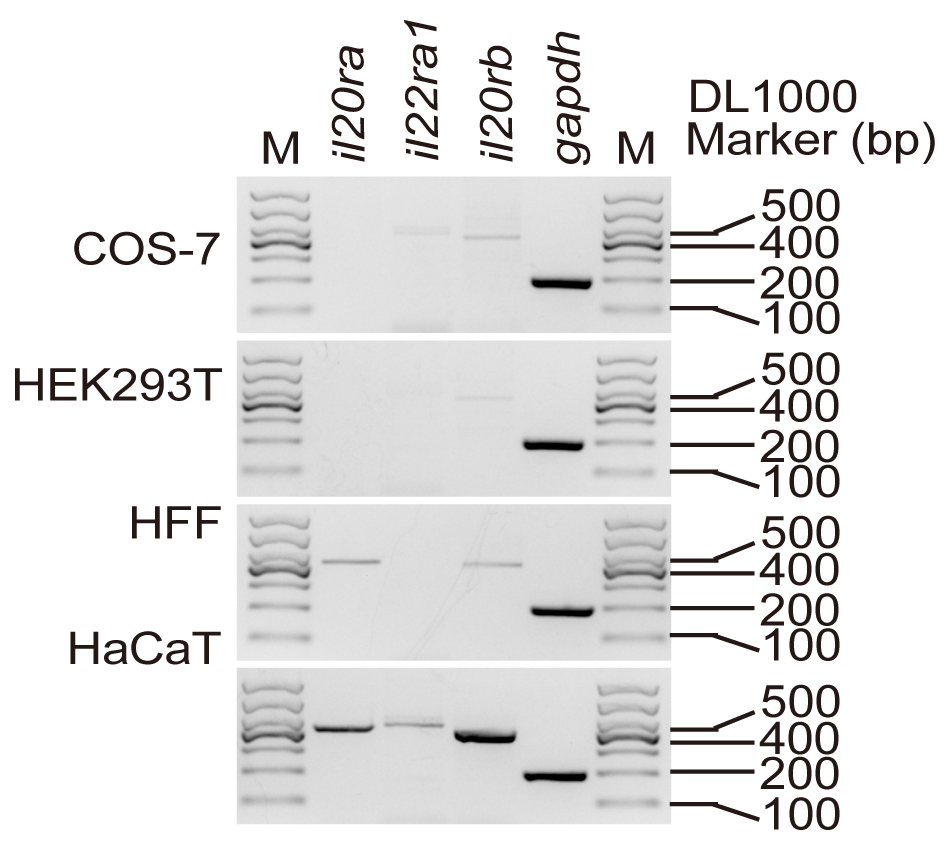

Supplement: Supplementary file 4 — Additional file 4: Figure S3. High transcription levels of IL20R subunits identification in HaCaT cells. Endogenous transcription levels of IL20RA, IL22RA1 and IL20RB in COS-7, HEK293T, HFF and HaCaT cells were detected with RT-PCR. [file 13071_2020_4251_MOESM4_ESM.tif]

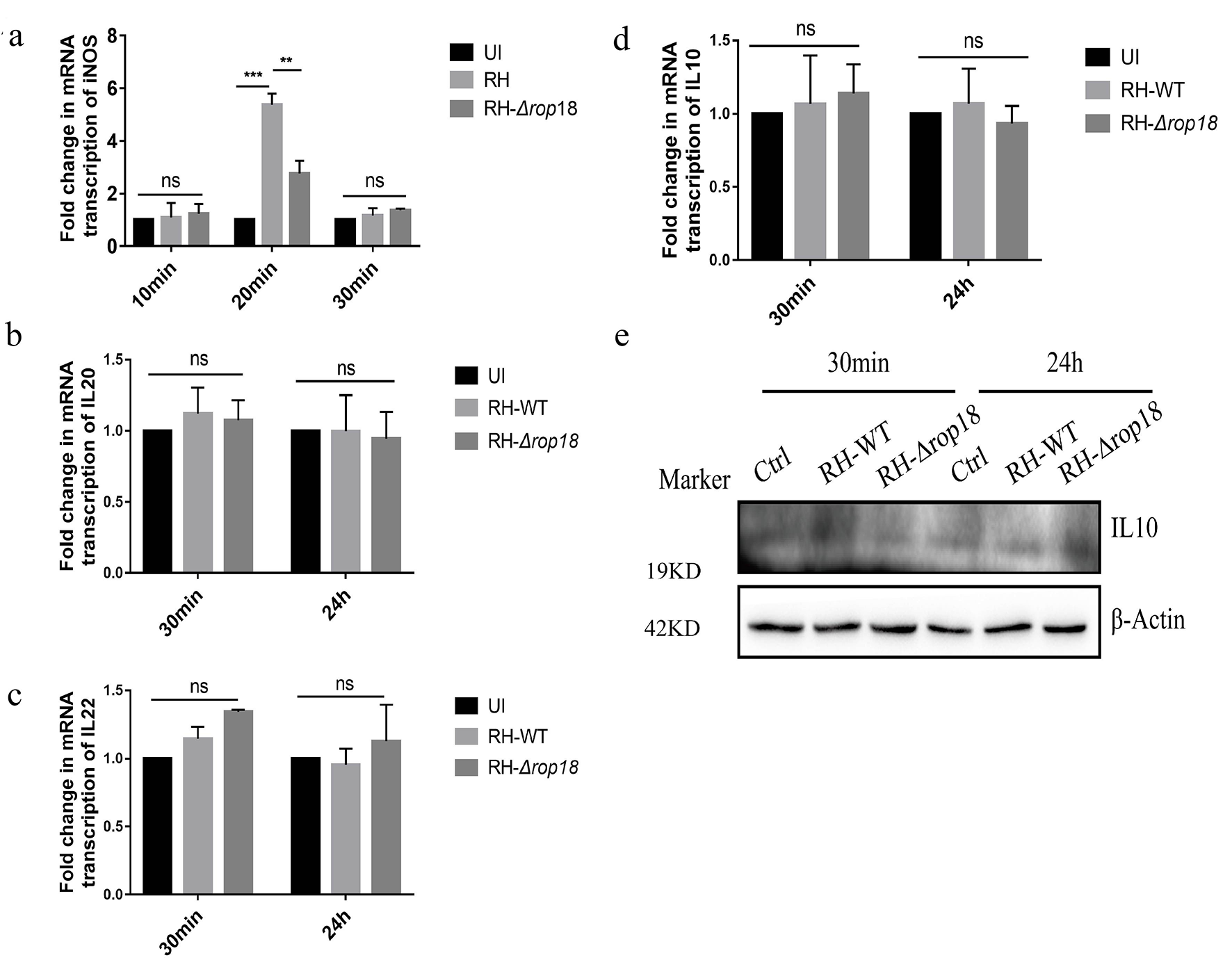

Supplement: Supplementary file 5 — Additional file 5: Figure S4. Comparison of the transcription and translation levels of iNOS, IL20, IL22 and IL10 in RH-WT or RH-∆rop18 infected HaCaT cells, and the uninfected cells. a iNOS transcription level in the RH infection group was significantly higher than in the other groups at 20 min post-infection, but not at 10, and 30 min (*P < 0.05). b-d No significant difference was found in the IL20, IL22, and IL10 transcription level, and the translation level of IL10 at both 30 min and 24 h post-infection among the indicated groups. [file 13071_2020_4251_MOESM5_ESM.tif]

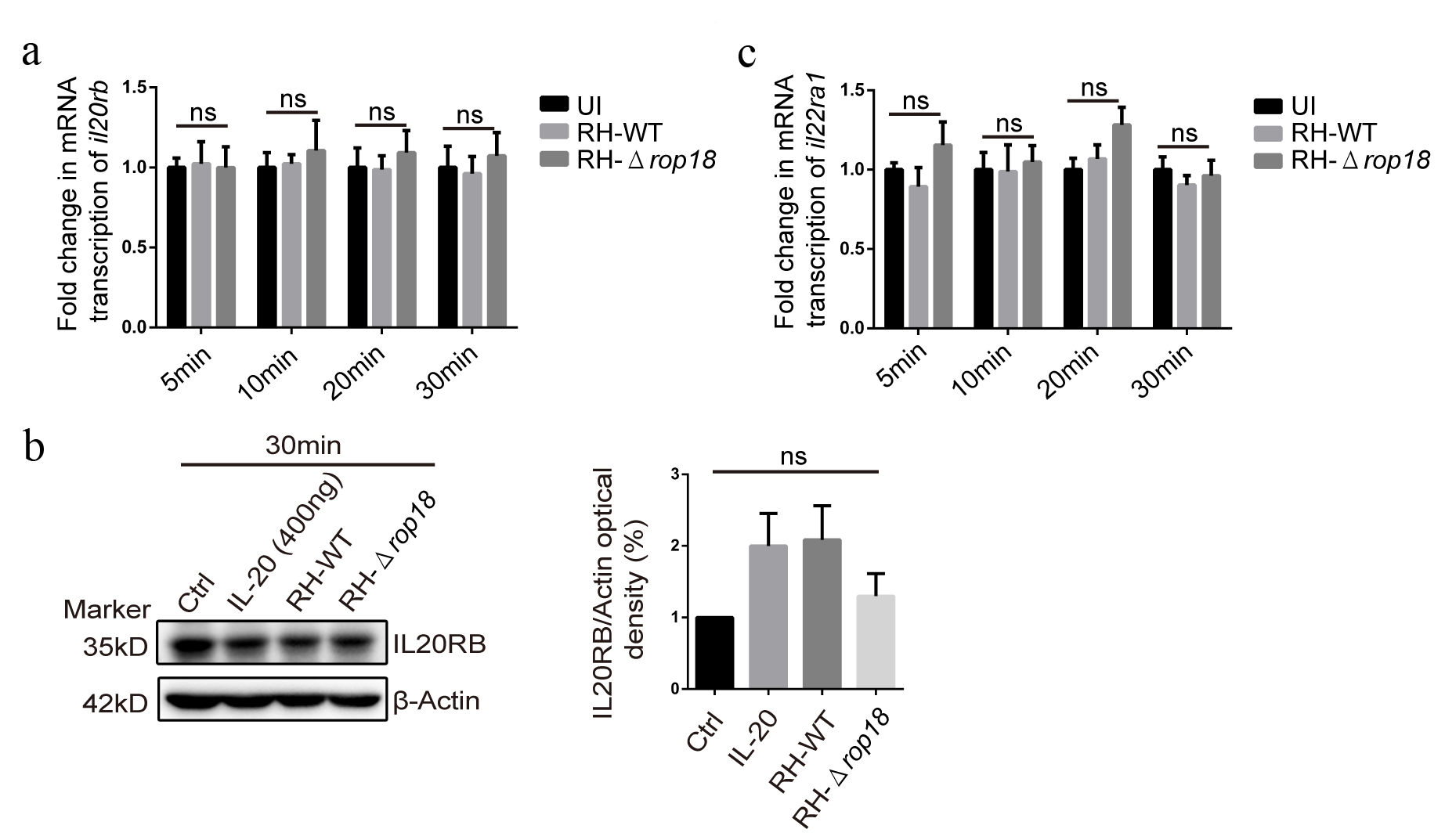

Supplement: Supplementary file 6 — Additional file 6: Figure S5.TgROP18 did not regulate the transcription and expression of IL20RB and IL22RA1. HaCaT cells were infected with indicated parasites for the indicated time. The transcriptional levels of IL20RB (a) and IL22RA1 (c) in HaCaT cells were detected with qRT-PCR. b The expression of IL20RB was detected by WB, no significant difference was found among the indicated groups (*P < 0.05). [file 13071_2020_4251_MOESM6_ESM.tif]
